# Machine Learning Based Prediction of Gamma Passing Rate for VMAT Radiotherapy Plans

**DOI:** 10.3390/jpm12122071

**Published:** 2022-12-15

**Authors:** Bartłomiej Sadowski, Karolina Milewska, Józef Ginter

**Affiliations:** 1 Medical Physics Department, The Maria Sklodowska-Curie National Research Institute of Oncology, 02-781 Warsaw, Poland; 2 Biomedical Physics Division, Institute of Experimental Physics, Faculty of Physics, University of Warsaw, 02-093 Warsaw, Poland

**Keywords:** gamma passing rate, patient specific QA, machine learning

## Abstract

The use of machine learning algorithms (ML) in radiotherapy is becoming increasingly popular. More and more groups are trying to apply ML in predicting the so-called gamma passing rate (GPR). Our team has developed a customized approach of using ML algorithms to predict global GPR for electronic portal imaging device (EPID) verification for dose different 2% and distance to agreement 2 mm criteria for VMAT dynamic plans. Plans will pass if the GPR is greater than 98%. The algorithm was learned and tested on anonymized clinical data from 13 months which resulted in more than 3000 treatment plans. The obtained results of GPR prediction are very interesting. Average specificity of the algorithm based on an ensemble of 50 decision tree regressors is 91.6% for our criteria. As a result, we can reduce the verification process by 50%. The novel approach described by our team can offer a new insight into the application of ML and neural networks in GPR prediction and dosimetry.

## 1. Introduction

Patient-specific quality assurance (PSQA) is a process recommended by many institutions, including the American Association of Physicists in Medicine (AAPM) [1]. It involves checking by independent methods the calculation or execution of a clinical plan created by a medical physicist on a specific treatment planning system (TPS). Verification of static plans involves checking with a second independent software (which uses, for example, a different calculation algorithm) [2].

Dynamic plans, on the other hand, are more complicated plans, and verification of such plans involves measuring them on a specific matrix [3,4]. Most often these matrices are expensive 2D arrays with ionization chambers or semiconductors. Such verification requires access to a linear accelerator beam, to configure and to calibrate detectors by a medical physicist. This process is time-consuming and leads not only to an extension of the patient’s start date (due to the wait time for the result of such verification), but also to additional costs incurred by the hospital for this type of verification, such as electricity and the purchase of the array [5].

The resulting dose distribution from the matrix is compared with the distribution calculated by the TPS and evaluated by gamma parameter [6].

In order to speed up the start of patient treatment, a machine learning algorithm could be created to predict the outcome of such verification. Several works have already been written on this subject [7,8,9,10]. In these papers, authors try to combine different types of coefficients for plan complexity, such as the modulation complexity score (MCS) [11], the number of monitor units, or the fractional dose to obtain a prediction of gamma parameter. This kind of method has a major advantage over ordinary software. The advantage would be that the software would give results based on plans that have already been verified early on (in other words, there will also be information on whether the treatment apparatus will execute the plan well). On the other hand, there are works that show that the problem of applying machine learning algorithms in PSQA is the quality of the data, more specifically, the small number of plans that do not pass the criteria of traditional verification or use parameters that in no way correlate with the gamma parameter [12]. This can lead to over or underestimation of results.

In this paper, we would like to present our results and a new approach using machine learning algorithms to predict the gamma passing rate parameter for the 2%/2 mm criteria for verification of therapy plans.

## 2. Materials and Methods

The data came from 13 months of work at the National Cancer Institute in Warsaw. VMAT treatment plans from the treatment planning system (TPS) Eclipse v15.6 (Varian, Palo Alto, CA, USA) for 1250 patients have been verified using an electronic portal imaging device (EPID) as1200 on TrueBeam accelerators (Varian, Palo Alto, CA, USA). The TPS prediction of fluency on the EPID was analyzed for the 2%/2 mm global gamma test [6]. For each plan, the gamma passing rate (GPR) was calculated using Portal Dosimetry v15.6 (Varian, Palo Alto, CA, USA]. For every patient, dosimetric analysis was performed separately for each arch. In total, there were 3166 arcs taken into account, treated hereinafter as separate plans. The data export and anonymization from ARIA OIS (Varian, Palo Alto, CA, USA) was automated using a script written in the AutoHotKey [13] language, which allowed to speed up and ensure the reliability of the data collection process.

To perform machine learning, various parameters were tested. For each plan, the input parameters were calculated and saved in the database, as well as the GPR value as an output parameter in the decision tree learning process. Machine learning was carried out in the Google Collaboratory environment [14] using the scikit-learn 1.0.2. library in Python 3.7.15. A decision tree regressor was trained to predict the GPR value. The impact of parameters on the outcome of GPR prediction by decision tree was analyzed using the “feature_importances_” parameter in scikit-learn.

Eventually, the 7 most significant parameters were left: MLC field, MLC field/Jaw field, average AAV, maximum AAV, average LSV, MU weighted LSV, and MCS. The explanations are given in Table 1.

Parameters such as AAV, maximum LSV, MU, fractional dose, and collimator angle were rejected as they were considered to be less important.

The vast majority of plans were above 99%. Only a few dozen plans had values below 98%. The proportion of plans with GPR (less than 97%: 97% to 98%, 98% to 99%: greater than 99%) was (23:41:181:2921). On this basis, we tried to develop and validate an algorithm to predict whether the newly prepared plan would achieve compliance between the fluency distribution predicted and measured by EPID with the GPR value above 98%.

The construction of the algorithm was finally based on a regression decision tree. To do this, two main problems had to be resolved. The first was the small size of the set of plans with a GPR value below the assumed threshold of 98%, which resulted in a lot of noise. For the same plan, the value was sometimes lower, sometimes higher. The second problem was a very unbalanced dataset. For the vast majority of plans, the dosimetric tests showed very high compliance with the predictions. Therefore, decision trees also tended to predict overestimated GPR values for all plans, including those with less agreement between the measured dose and the prediction.

In order to deal with the information noise, it was decided to train not just 1 tree, but a set of 50 decision trees, differing only in one parameter; the maximum depth when training successive trees ranged from 10 to 59. The squared error was adopted as the learning criterion. The number of features to consider when looking for the best split was 3, and the minimum number of samples required to be at a leaf node was 2. To deal with the excessive value predicted by the tree, a cut-off above the limit value of interest was applied: threshold = 98% + correction.

The parameter Σ has been introduced, equal to the sum of the answers below the cut-off threshold. It was hypothesized that plans for which Σ = 0 can be classified as meeting the criterion of high GPR value and did not require further dosimetric verification. Therefore, when analyzing the classification results, the following definitions were adopted: true positive (TP) are plans for which Σ = 0 and GPR ≥ 98%; false positive (FP) were those plans for which Σ = 0 and GPR < 98%; and true negative (TN) were those plans for which Σ > 0 and GPR < 98%. On this basis, it was possible to calculate the specificity $spec=\frac{TP}{TP+FN}$, which indicates the probability that the adopted course of action will not lead to the omission of the dosimetric verification of the plan with low compliance of the dose with the prediction. In addition, the probability of not skipping the verification of a plan with an actual GPR below 97% was also calculated, and we named it *spec97*. The ratio of TP to the number of all tested plans we named *gain*, because it shows what percentage of plans made in the hospital can be dispensed with dosimetry verification, thus gaining time.

In order to assess the effectiveness of the applied approach, the set of all data was randomly divided into a training part (2666 plans) and a test part (500 plans). This procedure has been repeated 1000 times. For each trial, the training of decision trees on the training set was performed, and the results of their predictions were analyzed on the test set.

### Gamma Passing Rate

The gamma parameter is used to compare the agreement of 2 dose distributions. The reference distribution results from the plan and the other distribution was measured using a matrix of detectors [15]. The gamma value is a quantity used to formulate a criterion for the deviation of the measured distribution from the reference distribution at a given point P on the EPID matrix.

To define this deviation, you must first adopt some values for 2 parameters: DD (dose difference) and DTA (distance to agreement). The first is expressed in percentages and the second in millimeters. The values of these parameters are adopted arbitrarily in accordance with the standards adopted in a specific medical center, typically it can be (DD, DTA) = (2%, 2 mm).

The distance $\gamma \left(P,M\right)$ is defined between a given point *P* with coordinates $\left(x_{P},y_{P}\right)$, where the planned percentage dose was $D_{P}$, and any point *M* with coordinates $\left(x_{M},y_{M}\right)$ and the measured percentage dose $D_{M}$. Its value is determined by the formula:

(1) $$\gamma \left(P,M\right)=\sqrt{\frac{{\left(x_{P}-x_{M}\right)}^{2}+{\left(y_{P}-y_{M}\right)}^{2}}{{\mathrm{DTA}}^{2}}+\frac{{\left(D_{P}-D_{M}\right)}^{2}}{{\mathrm{DD}}^{2}}}$$

This value can be thought of as a distance in the space of 3 coordinates *x*, *y*, *D*. The distance from point *P* in the reference distribution to the point M which is the nearest to it in the measured distribution is called the gamma value at point *P*:

(2) $$\gamma \left(P\right)=\underset{M}{\min}\gamma \left(P,M\right)$$

The dose value at a given point *P* on the EPID matrix is considered to have passed the test if $\gamma \left(P\right)<1$. The percentage of all points that pass the test is called the gamma passing rate. In this study, the so-called global gamma parameter was used, that is, the percentage dose was calculated as a percentage of the prescribed dose.

## 3. Results

**Figure 1:**
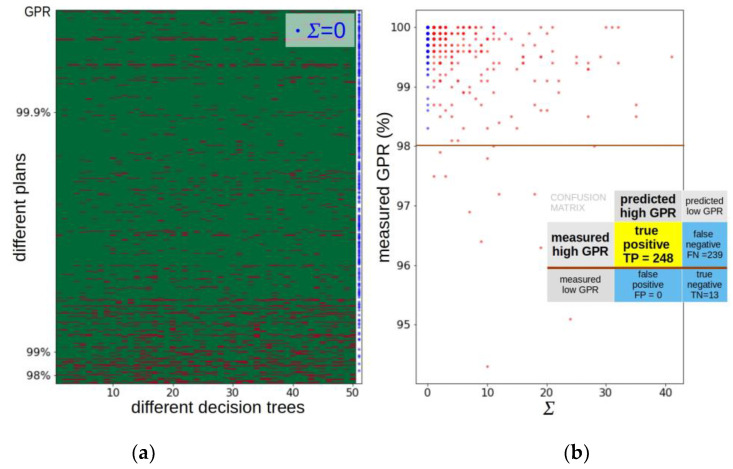
Example of prediction results. (**a**) Fifty trees (columns) tested on 500 plans (rows). Red boxes indicate values below the cut-off threshold (98 + 1)%. Blue dots on the right side mark the plans with no red boxes (Σ = 0). The plans are sorted by GPR, from smallest at the bottom to largest at the top. (**b**) The GPR value for each plan is presented as a function of Σ, with Σ = 0 dots in blue, and Σ > 0 in red. Due to the rounding, many dots overlap. The dots in the upper left corner represent half of all test plans.

Figure 1a shows the result of classification by different decision trees trained on a random sample of 2666 plans and tested on the remaining 500 plans. A cut-off threshold of (98 + 1)% has been used to predict the plans with a GPR over 98%. By convention, a fail (“predicted GPR below threshold”) is represented as a red rectangle and a pass (“we expect the GPR will be greater than the threshold”) is represented as a green rectangle. Each row in the diagram corresponds to one plan, with the plan placed higher the higher the measured GPR value was. Plans for which there was no red rectangle (Σ = 0) are marked with a blue dot on the right.

If the prediction accuracy of individual decision trees was high, one would expect that for plans above 98% most rectangles should be green, and for plans below 98% most should be red. The presented image does not look like this, and the distribution of green and red rectangles is quite chaotic. However, an order emerges from this chaos: there are clearly more red rectangles at the bottom of the diagram than at the top. For many plans no red rectangle appeared, and instead they were marked with a blue dot to the right.

The values of the parameter Σ were calculated for the entire test set. The results of the obtained dependence of the measured GPR value on the calculated Σ are presented in Figure 1b. One point on the plot corresponds to one plan, but due to the rounding of the GPR value to one decimal place, many points overlap. It is clear that while many plans with a high measured GPR have a value Σ of 0, all plans for which Σ ≥ 1 have a measured GPR significantly greater than 98%. The figure indicates the values from the confusion matrix for the answer to the question “will the measured GPR value exceed 98%?” based on the calculated value Σ for the sample test set. The obtained result suggests that if a plan results in Σ > 0, then we cannot say much about what GPR will be measured for this plan. However, in the opposite situation when Σ = 0, we can strongly conclude that the measured GPR value will exceed 98%.

In the example result presented, the probability that a plan with a low GPR value is classified as plans that do not require verification is described by $1-spec=\frac{FP}{FP+TN}=0$. In this case, the specificity is 100%. This is a very good result, but it is based on small statistics, because only 13 of all tested plans had a GPR < 98%.

**Figure 2:**
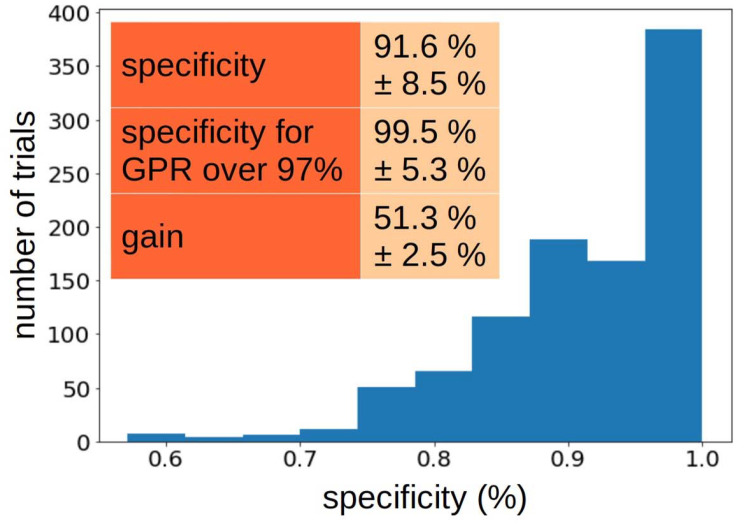
Histogram of the algorithm specificity in subsequent 1000 draws of data split into training and test sets. The average values of the prediction specificity of the GPR values for the tested 98% threshold, the average specificity for the 97% threshold, and the average time gain achievable with the proposed algorithm are given.

To estimate how good the proposed algorithm for finding plans with a high level of GPR is, the sampling of the training set and the training of the decision trees were repeated 1000 times, each time checking the specificity of the classification of the test set. The results are presented in the histogram in Figure 2. The mean specificity with standard deviation was 91.6% ± 8.5%. In order to determine the level of risk that the proposed algorithm will misclassify a plan with a GPR value significantly lower than the adopted threshold, the specificity for the answer to the question whether the GPR value for a given plan is greater than 97% was also estimated. The *spec97* value was 99.5 ± 5.3%. The average time gain that could be saved using this algorithm was 51.3% ± 5.3%.

In addition, the same values were also checked for the cut-off point (98 + 0.8)%. In this case, the *gain* increased to 0.60, but the average specificity dropped to 0.87, and the *spec97* value dropped to 98.9.

## 4. Discussion

Many papers have previously to find a way to predict the GPR value based on the calculated parameters of the plan. For this purpose, various coefficients of plan complexity were constructed, various machine learning algorithms were used, and in recent years, deep learning has also been implemented. Against the background of these works, our algorithm seems relatively simple. It also seems quite effective from the point of view of saving staff time and costs related to the use of the accelerator in the hospital. If we accept the specificity of prediction at a level of over 90%, this method would make it possible to resign from the dosimetry test on average for every second plan. For example, if during day 20 such tests are performed in a hospital, which corresponds to two hours of measurements, based on the proposed algorithm, 10 of them could be abandoned, which means saving one hour of accelerator use per day. While this may lead to mistakes, we estimate the probability that a clearly inferior plan will be misclassified to be less than 1%.

In the described approach, it seems to give a good result to use a set of weak classification algorithms instead of looking for one very good one. Each of them learned to pay attention to some feature in the training data. Interestingly, although the subjective viewpoint of one decision tree did not lead to satisfactory conclusions, it turned out that simply combining many subjective viewpoints resulted in a method for making good decisions.

An arbitrary cut-off threshold (98 + 1)% = 99% was used in the way of defining the parameter Σ. It was then checked by repeated randomization of different divisions into the training and test sets, to determine the average effect of such an approach on the process of dosimetry of plans in the hospital. Although the number of low-GPR plans in our case was small, the reasoning behind the bootstrap technique suggests that the histogram shown in Figure 2 may be a good representation of how the algorithm would perform if we trained the algorithm on the entire dataset we have and used it with other plans prepared at the National Cancer Institute, not included in this study. The obtained specificity of 91% is the expected value, but there is a probability (proportional to the value in the histogram) that the algorithm would handle the data better or worse. Therefore, the achieved result could not be applied in practice without additional tests. Furthermore, as new data are obtained, the algorithm should be successively calibrated.

For the cut-off value used for the regression results, we obtained the presented results, but it is a matter of debate whether the value of this parameter used was optimal. In this respect, we are dealing with a trade-off. Setting this value lower leads to an increase in time gain at the expense of a decrease in specificity, which increases the likelihood of a mistake. It seems that in clinical practice this should be avoided. On the contrary, increasing the cut-off threshold would result in an algorithm that is less error-prone, but also less time-saving.

The studies described are preliminary results. We believe that researchers with better data than ours (more numerous and more diverse in terms of measured GPR values) could easily obtain better results with this method. This will also be the subject of further research by our team.

## Figures and Tables

**Table 1:** Significant parameters included in the machine learning algorithm.

Parameter	Definitions
MLC field	Sum after control points of field sizes determined by MLC.
MLC field/Jaw field	The sum of the ratios after the checkpoints of the sizes of the fields determined by the jaws and MLCs.
Average AAV	Average value of aperture area variability (AAV), or in other words, the area of the beam aperture. AAV is a component of MCS. Calculation of AAV is described in [11].
Maximum AAV	Maximum value of aperture area variability.
Average LSV	Average value of leaf sequence variability. This parameter is defined to characterize the variability segment shape for a specific plan. LSV also is a component of MCS, described in [11].
MU weighted LSV	Value of LSV weighted by monitor units.
MCS	The modulation complexity score (MCS) is a metric used to characterize a treatment plan. MCS includes information such as: variability in leaf position, degree of irregularity in field shape, segment weight, and area. The value of MCS is between 0 and 1. The leaf sequence variability (LSV) and aperture area variability (AA) are combined to calculate MCS. Calculation of MCS is not within the scope of this article, MCS is described in [11].

## Data Availability

Not applicable.
